# Coherent-Phase Optical Time Domain Reflectometry for Monitoring High-Temperature Superconducting Magnet Systems

**DOI:** 10.3390/s25237368

**Published:** 2025-12-03

**Authors:** Matthew Leoschke, William Lo, Victor Yartsev, Steven Derek Rountree, Steve Cole, Federico Scurti

**Affiliations:** 1 Ken and Mary Alice Lindquist Department of Nuclear Engineering, The Pennsylvania State University, 205 Hallowell Building, University Park, PA 16802, USA; mpl5924@psu.edu (M.L.); williamlo@psu.edu (W.L.); 2 Luna Innovations Inc., 3155 State Street, Blacksburg, VA 24060, USA; victor.yartsev@lunainc.com (V.Y.); rountreed@lunainc.com (S.D.R.); stevepcole@icloud.com (S.C.)

**Keywords:** optical fibers, Rayleigh scattering, distributed temperature sensing, high-temperature superconductors, quench detection, fusion

## Abstract

High-temperature superconductor (HTS) magnet systems, especially those designed for fusion reactors, require effective and reliable monitoring to avoid damaging anomalies. In tokamaks, some of the magnetic coils are time-dependent, which causes strain and large inductive voltages within the magnet, rendering detection of incipient quench challenging. Ionizing radiation can also create material defects and lead to non-uniform degradation of conductors. The resulting decrease in critical current uniformity across the magnet, along with manufacturing defects, such as failure of structural materials or cooling systems, can all potentially initiate a quench. HTS magnets have a lower normal zone propagation velocity than low-temperature superconductors, and this causes normal zones to be localized, increasing the risk of permanent damage. Fiber optic sensors have several qualities that are essential in fusion systems. Unlike traditional voltage-based sensors, fiber optic cables are immune to the large electromagnetic fields present. This study presents and validates a fiber optic interrogation technique for monitoring magnetic confinement fusion and other high-temperature superconducting magnet systems. Coherent-phase optical time domain reflectometry (OTDR) allows for the high sampling rates (tens of kHz) necessary to quickly detect and mitigate quench events over the long distances required to monitor fusion magnet systems. This technique was demonstrated to successfully detect localized thermal transients at cryogenic temperatures as low as 6 K. These outcomes were also demonstrated using fibers embedded in HTS magnet coils at 77 K, verifying the potential for this interrogation technique’s use for failure detection in HTS coils.

## 1. Introduction

Superconducting magnets are being explored for a variety of applications, including plasma fusion containment systems, medical imaging, magnetic levitation transport, degaussing, and novel computing hardware. High-temperature superconductor (HTS) magnets are particularly compelling for magnetic confinement fusion because of their ability to generate high magnetic fields, allowing for more compact reactor designs [1,2,3,4,5,6]. Precise control of superconducting magnet systems is key to ensuring safe, stable operation [7,8,9,10,11,12,13]. Quench events, where a portion of the magnet loses its superconducting properties, can damage a superconducting magnet system if not detected quickly, allowing for corrective action to be taken. Large amounts of energy can be stored in the magnet field; when rapidly converted to heat, the magnet may be damaged. In addition, heat will increase the surrounding temperature, possibly causing the quench to propagate to other sections of the magnet as they leave the superconducting state. Detection systems must exist to rapidly identify temperatures at specific locations over the extensive lengths of HTS magnet conductors, which can be several kilometers. Control devices need to be able to accurately record under high magnetic fields and cryogenic temperatures. In some applications, such as nuclear fusion reactors and particle accelerators, the presence of ionizing radiation further complicates the local environment for sensing. Recently, the effects of ionizing radiation on optical fibers have been studied in the context of their integration into nuclear fusion reactors [14,15,16,17,18]. These efforts aimed to assess the radiation tolerance of fiber sensors for temperature measurements [19], mitigate undesired radiation-induced attenuation [20], and exploit radiation effects for measuring doses at cryogenic temperatures in fusion environments [21].

For the aforementioned reasons, several magnet monitoring and fault detection systems based on various techniques are under development [22]. In addition to optical fiber sensors, other methods are based on acoustic signals [23,24], magnetic field changes [25,26], capacitance changes [27,28], and RF signals [29,30]. Measurement solutions based on embedded optical fibers have the advantage of being immune to electromagnetic noise, a property that makes them an attractive alternative to voltage-based systems. Fiber optic measurement techniques rely on tracking the spectral change in an optical signal sent through the fiber from a remote interrogator. In most techniques, applied changes in temperature and strain affect the fiber’s material properties and/or strain state, resulting in a modification of the reflected signal. High acquisition rates are essential for monitoring HTS magnets in real time, enabling control systems to respond quickly and mitigate quench events before they spread. Optical sensors that have a high temporal resolution include the Fiber Bragg grating (FBG) and Fabry–Pérot Interferometer (FPI) [31,32,33]. However, these are point sensors that cannot perform distributed measurements individually. While gratings can be combined on a single fiber [34], there are limits to the spatial resolution and range of this method. These can be useful as a method of calibration for the distributed sensing system due to their accuracy and high temporal resolution.

Rayleigh backscattering is an optical phenomenon that provides a basis for continuous fiber optic sensing, observing conditions at all locations on a fiber. The material in the core of an optical fiber is not uniform when created, and slight imperfections cause variations in the density of the glass. Light traveling through a fiber will scatter at these unique scattering centers, with some returning in the opposite direction along the fiber. While this loss is an issue for transmission in telecommunications, it can be used to determine properties of the fiber based on location. While Rayleigh-based sensing utilizes elastic scattering, the inelastic Brillouin and Raman scattering can also be employed for distributed sensing. Brillouin Optical Time Domain Analysis (BOTDA) can interrogate over long lengths of fiber, but this is limited by spatial resolution and measurement time. Currently, BOTDA is not able to produce an accurate measurement within less than 0.5 s; this is especially challenging at cryogenic temperatures. Raman scattering has been considered for HTS quench detection [35]; however, it has low to no sensitivity below 70 K and is therefore unsuitable for fusion magnet systems and most superconducting magnets.

**Figure 1:**
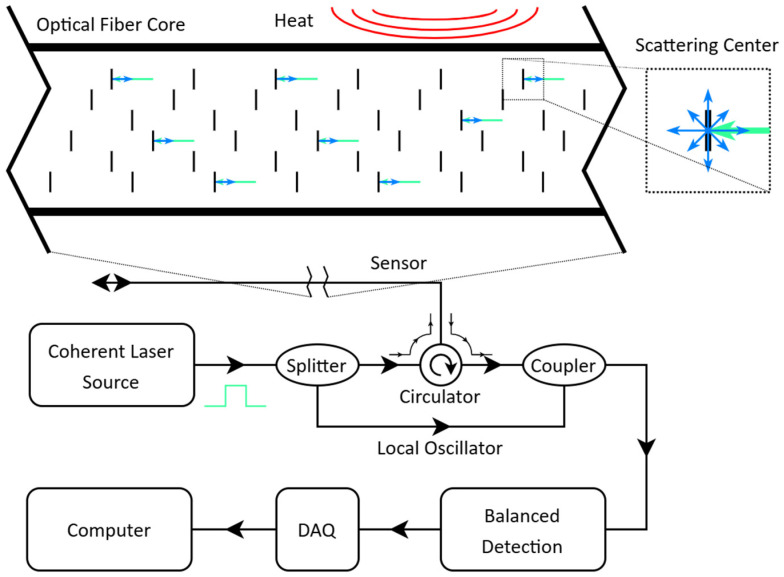
Diagram of coherent-phase OTDR interrogation process, as well as a microscopic view of Rayleigh scattering interactions within the fiber core.

Optical time domain reflectometry (OTDR) is a method of testing the quality of fiber over long distances, locating losses based on the amplitude of scattered light. A laser emits a pulse through the length of the fiber, and location can be determined by the time it takes for backscattered light to return to a detector parallel to the source (Figure 1). This uses the fiber as a continuous sensor that can be divided up into sections of length based on time of flight, quantified as gauge length. The rate of each pulse is based on the length of the fiber, as the interrogator must wait for all possible light to be reflected to avoid interference between pulses.

A widespread Rayleigh-based technique is optical frequency domain reflectometry (OFDR), which analyzes data in the frequency domain, enabling high spatial resolution. In the past, research has focused on this method for distributed sensing within superconducting magnets [7,8,9,10,11,12,13]. However, as data must be transformed to the frequency domain, this method is computationally intensive and thus has lower temporal resolution, as well as limited spatial range. The temporal resolution is additionally limited by the fact that OFDR requires the laser wavelength to be swept across a wide range, compared to a simple coherent pulsed laser used in OTDR. It is also challenging to maximize fiber length and measurement rate. Despite the fiber length limitation, this prior work using OFDR to interrogate optical fibers showed that sensing temperature and strain is advantageous in HTS compared to voltage [7], that the temperature and strain changes can be decoupled in a HTS coil regardless of the sensing technique [8], and that the optical fiber can be successfully integrated into HTS wire [10] and multi-wire HTS cables [11] and in coil-to-coil insulation [12]. These successes, which are virtually completely independent of the sensing technique, motivated this work into other sensing techniques that could overcome the fiber length and interrogation speed limitations of OFDR. While the techniques are both based on the same scattering process, OTDR has the ability to operate at drastically higher measurement rates and fiber lengths, on the order of tens of kilohertz and tens of kilometers, respectively. The third parameter, spatial resolution, is demonstrated in this work to not be significantly limiting for quench detection, even if it is beneath that of OFDR.

A high-coherence laser source allows OTDR to become a continuous distributed sensor; the phase of the backscattered light can be measured due to the high coherence of the source, and this phase is sensitive to changes in optical path length caused by vibration, strain, and temperature. The capabilities of coherent-phase OTDR have been demonstrated in geophysical applications at room temperature [36,37,38,39,40]. The highly stable light source used in coherent-phase OTDR can capture minute changes in optical path length corresponding to nanometer-scale displacements (Figure 1). An excellent review of the technique can be found in [41]. Distributed temperature measurement using coherent-phase OTDR was previously demonstrated at and around room temperature (for temperature changes up to 10 °C within the 50–90 °C range) [40], showing that the low-frequency portion of the acquired signal scales with temperature. This prior work further motivates this study, which focuses on the cryogenic temperature range. In addition, there have been previous studies on HTS quench detection using a similar, phase-sensitive OTDR sensing technique using a different interrogator that measured equivalent strain instead of phase [14,42]. These experiments also characterized cryogenic temperatures using a cryocooler, though with a different gauge length, channel spacing, acquisition frequency, and fiber integration with HTS wire, and were able to show promise for cryogenic temperature profiling.

This study investigates a system for the continuous monitoring of large-scale infrastructure in magnetically confined fusion reactors via embedded fiber optic cables through coherent-phase OTDR. Its features are highly suited to the rapid detection of localized heating indicative of incipient catastrophic failure in HTS magnets. OTDR does not require transformation to the frequency domain, which is a temporally costly aspect of OFDR data processing. This method has the potential to provide undisturbed and simultaneous measurements of minute strain and temperature variations over tens of kilometers and up to sampling rates of 40 kHz. It can produce a spatial resolution as small as 40 cm; while this cannot compare to that of OFDR, this study affirms that it is sufficient for the purpose of hotspot detection. Considering its high temporal sampling rate and long range, the coherent-phase OTDR technique offers the potential to detect quench events in fusion reactors much more quickly than other techniques, while covering the entirety of the magnet system. In the following experimental study, the viability of the coherent-phase OTDR technique in detecting thermal transients at cryogenic temperatures is investigated.

## 2. Materials and Methods

**Figure 2:**
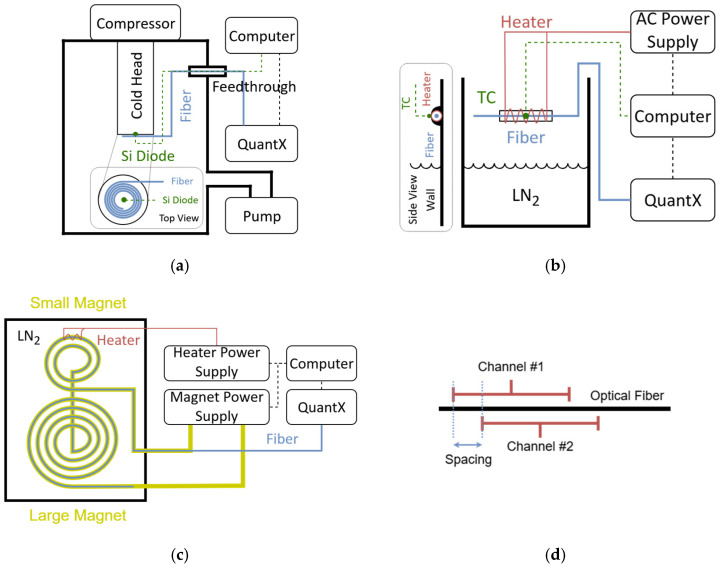
Experimental setups for (**a**) cryocooler, (**b**) heater, and (**c**) magnet testing. A diagram indicating channel length and spacing (**d**) is also shown.

A series of experiments were conducted using a OptaSense QuantX Distributed Acoustic Sensing interrogator, Luna Innovations, Roanoke, VA, USA to sense fiber optic cables at ambient and cryogenic temperatures in a variety of settings with the coherent-phase OTDR technique. Figure 2 shows experimental diagrams of the cryocooler experiment, heater tests in nitrogen vapor, and magnetic coil setup.

The first series of tests, looking at localized heat recognition, were conducted using a polyimide-coated Ge-doped single mode fiber optic cable with a small wire heater at a single location on the fiber. Tests were conducted at room temperature, as well as at cryogenic temperatures by attaching the fiber above liquid nitrogen in a Styrofoam cooler. Different amounts of heat, from 0.5 W to 5.0 W, were applied over 20 s intervals to a 5 cm long cylindrical wire heater attached to the wall of the cooler. The fiber was threaded through the center of this metal cylinder, such that the heat was applied to 5 cm of fiber. Temperature was measured using a Type K thermocouple (attached to the surface of the cylinder) in parallel to the measuring phase with the QuantX interrogator. Each channel (gauge) corresponds to the average response over a 2 m long fiber segment, spaced 40 cm apart (Figure 2d). The sampling rate for each test was 40 kHz. These interrogator settings were applied to subsequent tests as well.

Cryogenic testing was then performed solely examining the QuantX response with fibers in the cryogenic temperature range. The second series of tests looked at the response of the system to gradual temperature changes in the fiber over multiple hours covering a wide range of temperatures, to understand the relationship between measured phase and temperature. A loop (3.5 m long, 40 cm diameter) of polyimide-coated Ge-doped single mode fiber was attached to the stage (65 mm diameter) of the Sumitomo cryocooler cold head and connected to the QuantX interrogator via a 9 m lead fiber. The cryostat containing the cold head was evacuated to a pressure of about 7 µbar. The temperature was conductively lowered from room temperature to the minimum temperature of the cryocooler, 6 K, where temperature was held constant for 20–30 min. The cryocooler was then turned off and the fiber was allowed to heat up slowly to room temperature.

Once the coherent-phase OTDR technique was proven in isolation, experiments were conducted using an optical fiber integrated in an HTS magnet in liquid nitrogen. This was performed in order to demonstrate that the interrogator was capable of detecting and locating changes in temperature in a superconducting magnet during current flow and under a magnetic field. An acrylate-coated (10 μm) reduced-cladding (80 μm) single mode Ge-doped fiber was wound with HTS tape in a dual coil magnet system: one 30 m long pancake coil connected to one 70 m long pancake coil. Direct current was increased at a rate of 3 A/s for 15 s, held constant at 45 A for 40 s, and then gradually reduced to zero. During the 40 s that current was constant, heat was applied for the second set of 10 s. This test was repeated multiple times, with an embedded wire heater producing powers of 0.5 and 1.0 W. Currents and heater powers were chosen such that the magnets would not actually quench in this experiment, to assess whether a smaller amount of heat than what is needed to damage the magnet could be detected. These tests were conducted by immersing the magnet coil system in liquid nitrogen using a cryostat.

Confidence intervals (99%) are included for each of the three tests performed to quantify statistical error of the phase response. For the localized heater tests, uncertainty was determined for a rolling 4 s window after detrending using a Savitzky–Golay filter. Error in the cryocooler and magnet tests were computed using standard deviation of multiple tests. For plots where phase is derived from temperature data, error values for the Si diode (±100 mK) and thermocouple (±250 mK) were summed in quadrature.

## 3. Results and Discussion

**Figure 3:**
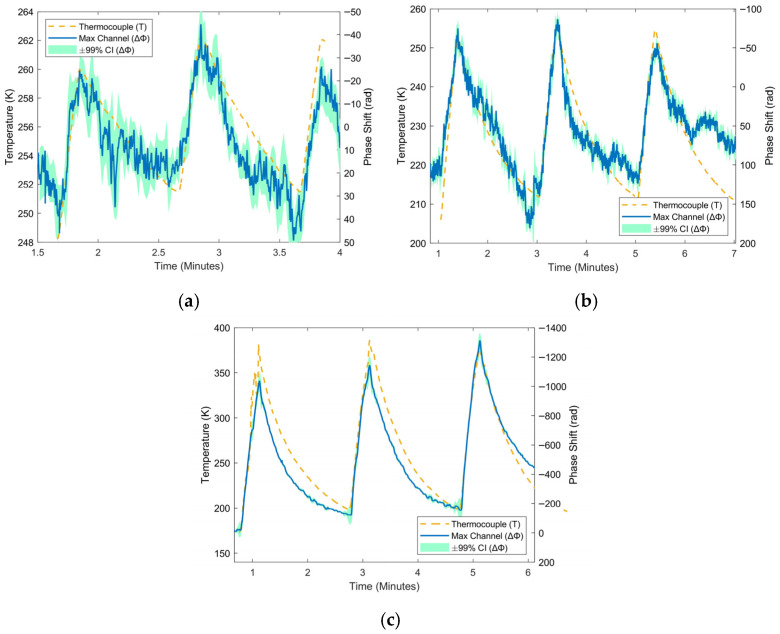
Response of fiber to localized heating at various cryogenic temperatures using a 5 cm heater. Three heating powers, each applied over 20 s, are compared here: (**a**) 0.5 W, (**b**) 1.0 W, and (**c**) 5.0 W. The blue curve shows the phase measured by the system, while the yellow curve shows temperature measured by a thermocouple.

The first set of results are from the heater tests in nitrogen vapor, where a 5 cm copper tube wrapped in nichrome wire was heated with varying amounts of power in pulses. Phase shift has a clear response to the introduced heat, as shown in Figure 3. The phase response closely tracks the response produced by a thermocouple located at the center of the heater, and this response also scales with the thermocouple as heater power increases. Table 1 summarizes values for SNR and temperature sensitivity as a function of heater power. Noise does not appear to increase at higher powers, as SNR values generally increase with heater power. Phase sensitivity to temperature also decreases as temperature increases, a trend that matches the characteristic curve we have measured.

An important finding relates to the physical dimension of the heated region: the fiber section that was covered by the heater was only 5 cm, or 2.5% of the 2 m channel length. The ability to detect and track such a highly localized temperature change is of great promise for HTS magnet applications, due to the slow speed of normal zone propagation. This low speed causes heat to build up in localized hotspots, and high spatial resolution is required to determine their location before damage occurs. These results indicate that a relatively long channel length of 2 m could be sufficient in most applications, as opposed to the very short channel length of a few millimeters previously used in optical fibers interrogated by OFDR in superconducting magnet applications [7,8,9,10,11]. The spatial resolution of this technique is on the order of the meter, while channel spacing can be below 10 cm; in our case, the gauge length was 2 m with 40 cm spacing. Spatial resolution depends directly on pulse width, or the duration of the light pulse launched into the fiber. Higher spatial resolution can be achieved at the expense of SNR by varying the discretization and digitizer sampling rate. The 40 cm spacing was chosen to optimize these factors, as well as prevent data volumes from becoming unreasonably large with the 40 kHz sampling rate. At this sampling rate, the amount of data collected over time periods of several minutes to several hours can become difficult to process without a dedicated computational infrastructure. In the future, this trade-off can be determined more systematically, which will likely require modeling work to minimize the number of experiments as a function of operating temperature, current margin, external field, and other factors.

Compared to OFDR, the main limitation is the averaging effect over the gauge length, which can reduce sensitivity to highly localized hotspots. For example, a uniform temperature rise in ΔT across the full gauge would produce a large phase shift, while the same ΔT confined to only part of the fiber gauge length would yield a much smaller signal, because only a fraction of the fiber segment contributes to the phase shift. Since hotspots in HTS magnets can be as small as a few centimeters wide, this represents the most challenging detection scenario. To test the ability of the technique to detect highly localized hotspots in this worst-case scenario, we applied 5 cm heat pulses (2.5% of the 2 m gauge length) and successfully detected the resulting temperature rise, demonstrating the technique’s ability to identify hotspots despite their small size relative to the gauge length.

**Figure 4:**
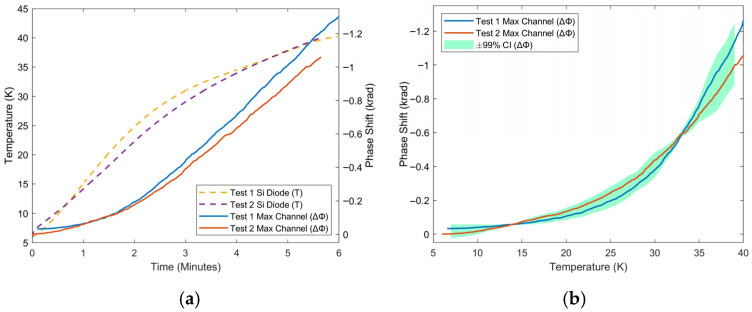
Response of maximum channel phase to warm-up of cryocooler between 6 K and 40 K as a function of (**a**) time and (**b**) temperature for repeated tests. The solid curves show the phase measured by the system, while the dashed curves represent temperature measured by a silicon diode.

**Figure 5:**
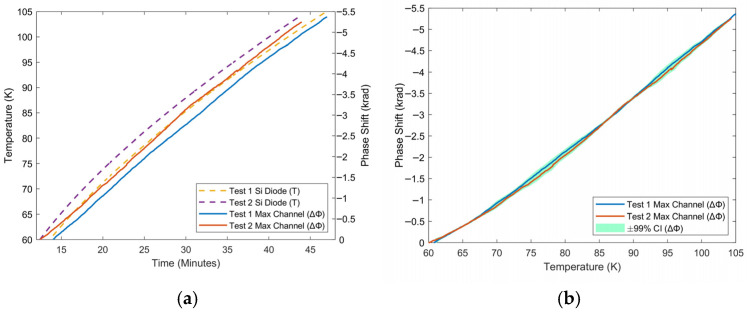
Response of maximum channel phase to warm-up of cryocooler between 60 K and 105 K as a function of (**a**) time and (**b**) temperature for repeated tests. The solid curves show the phase measured by the system, while the dashed curves represent temperature measured by a silicon diode.

**Figure 6:**
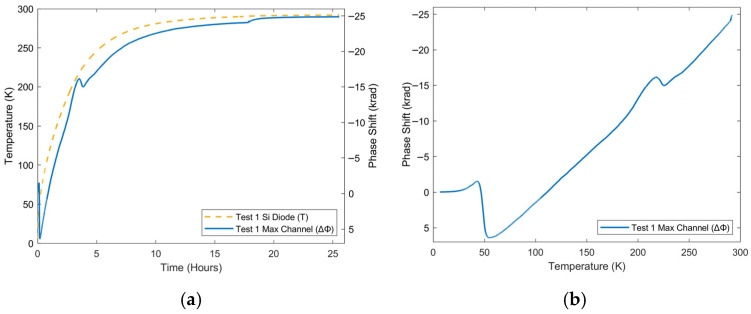
Response of maximum channel phase to warm-up of cryocooler between 60 K and 293 K as a function of (**a**) time and (**b**) temperature for one test. The solid curve shows the phase measured by the system, while the dashed curve represents temperature measured by a silicon diode.

Cryocooler tests produced a promising phase temperature curve in the cryogenic temperature region. For these tests, data is presented from immediately after the cryocooler compressor is turned off, up until room temperature is reached. The channel shown has the maximum phase response, corresponding to the section of fiber positioned on the coldest part of the cryocooler’s cold head. Above 60 K, there is generally an inverse linear correlation between temperature and phase; below this temperature, sensitivity starts to decrease. Figure 4, Figure 5 and Figure 6 show this response between 6 and 40 K, 60–105 K, and 6–293 K. In the low-temperature cryogenic range, from 6 to 40 K (Figure 4), there is a monotonic response in phase as temperature increases. Despite being the lowest in this range, temperature sensitivity is on average 10 rad/K even at the lowest temperatures (6–25 K), and sensitivity continues to increase as a function of temperature. This sensitivity is orders of magnitude over that which was seen in the heater tests, and this can be explained by how well the fiber was coupled to the surface of the cold head, via vacuum grease in a low-pressure environment. By increasing contact between the fiber and the object being measured, sensitivity can be increased. Additionally, this section demonstrates repeatable trend results over two tests. In the high-temperature cryogenic range, from 60 to 105 K (Figure 5), there is a visibly linear response to temperature, with only slight variations. There is also high repeatability between the two tests.

The minimum detectable temperature change, or temperature resolution, was found to be 0.9 K at 20 K with a 68% confidence interval (1.8 K with a 95% CI). As expected, the temperature resolution improved at higher temperatures. At 30 K it was found to be 0.55 K with a 68% CI (or 1.1 K with a 95% CI).

These favorable results of temperature resolution and repeatability are significant for application to HTS magnets that operate in this temperature region. A final section, presenting the entire range of the test, from 6 K up to room temperature, is shown in Figure 6. Overall, the phase response over this period at higher temperatures is nearly linear in relation to the Si diode response (Figure 6b), barring an artifact near 215 K. As the dominant method of heat transfer is conduction, the rate of temperature change is proportional to the temperature difference between the fiber and the surrounding environment, via the cold head. This trend of positive exponential decay to room temperature is reflected in both responses. In general, phase has a similar response to a silicon diode for temperature measurement during this characterization of phase response between 6 K and room temperature and is repeatable. This is especially true during the high-temperature cryogenic range applicable for HTS magnets (60–105 K), as the phase-temperature curve becomes linear.

**Figure 7:**
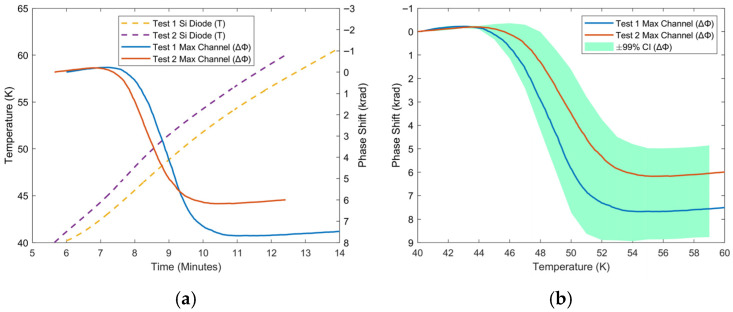
Response of maximum channel phase to warm-up of cryocooler between 40 K and 60 K as a function of (**a**) time and (**b**) temperature for repeated tests. The solid curves show the phase measured by the system, while the dashed curves represent temperature measured by a silicon diode.

**Figure 8:**
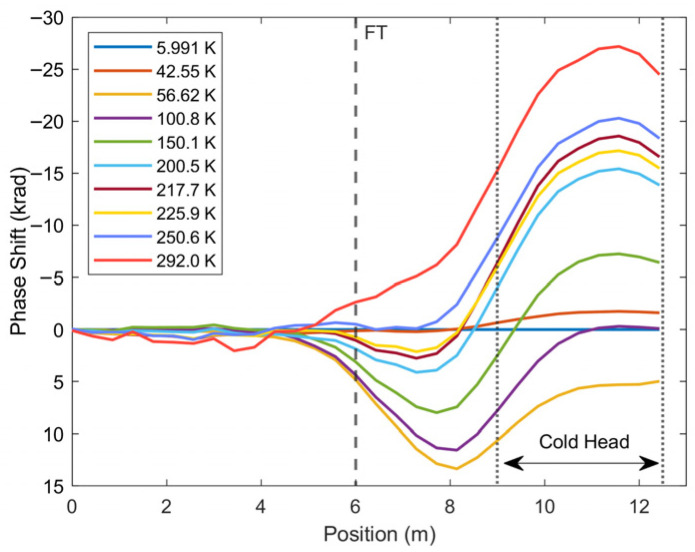
Spatial distribution of phase at multiple temperatures during warm-up of cryocooler (test 1). The fiber enters the low-pressure chamber containing the cryocooler through a feedthrough (FT). It is then coiled on the cold head, where the minimum temperature occurs.

Despite this, there are two deviations from this trend that should be noted: a prominent reversal in phase direction between 40 and 60 K, shown in Figure 7, and a smaller reversal from 215 to 230 K. The phase response should correspond to a decrease in temperature, which is not reflected in the silicon diode in either case. Moreover, the larger reversal occurs in both tests 1 and 2, implying that this was not an artifact of a single test, unrelated to the experiment. The smaller reversal was also seen to be repeated in other tests. The spatial distribution of phase (Figure 8) may provide an explanation for these deviations from the temperature trend. This figure displays the phase response outside of the low-pressure cryostat chamber (0–6 m) having little to no response, as this section is at room temperature. There is also a response that mirrors the temperature distribution, centered on the section of fiber on the cold head (9–12.5 m), which warms up from 6 K to room temperature, consistent with expectations. However, there is an unexpected distribution centered on the section of fiber between the entrance to the cryostat and the cold head, most of which was taped in a loop. This section seems to be the source of the two anomalies unrelated to temperature.

These deviations appear to be related to the setup of the experiment, as the anomaly from 215 to 230 K was not observed in the nitrogen vapor heater experiments. The low temperatures of this experiment occur via a low-pressure system, and during the warm-up, temperature and pressure increase after the compressor is turned off. A phenomenon that would explain a repeated response at a specific temperature over multiple experiments would be a vibrational response to a phase change. Higher temperatures outside of the cold head, where the silicon diode is not recording, in combination with low pressure allowing for sublimation at lower temperatures, would lead to the conclusion that this phase change is the sublimation of nitrogen. Looser sections of the fiber will vibrate as nitrogen that has deposited on the fiber returns to vapor, as well as due to general pressure changes in the chamber. This interrogation method seems to be more sensitive to vibration in comparison to temperature, which would explain why phase drops below the initial phase at 6 K. This may also cause strain between loose sections and sections that are taped down; however, this effect would not be as clearly repeatable as is shown in the data and would not occur on the same time scale. The smaller reversal at 215 K may be attributed to the sublimation of water vapor. While future work will clarify the source of these deviations, it is clear from these datasets that they are occurring outside of the cold head and only their tail is influencing the signal measured on the cold head. This also highlights that the vibrational sensitivity of this interrogation must be investigated in relation to temperature sensing.

The separation of vibrational and strain effects from temperature responses will be key in the use of this interrogation method for HTS quench detection. While this initial data shows promise for the sensing technique, future studies on this technique will explore cross-sensitivities at cryogenic temperatures and its application in HTS magnet quench detection. A possible option to achieve this would be training a machine learning classifier to distinguish between localized heating and vibrations. Another method would rely on acoustic signals and determine changes in acoustic propagation caused by temperature variation [23,24]. This could also be performed physically by outfitting the optical fiber with a jacket to provide vibration damping and thus enhancing the temperature signal.

**Figure 9:**
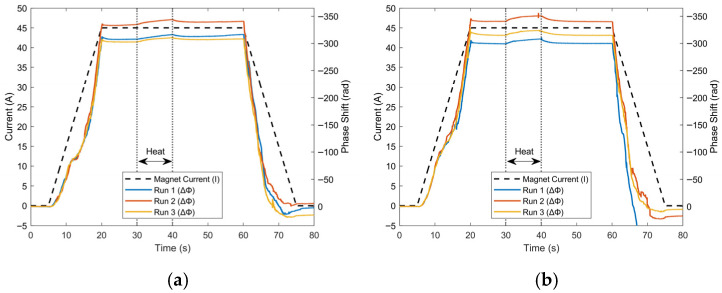
In-magnet test phase and average transport current as a function of time for three consecutive runs where current and heat were applied for each heating power: (**a**) 0.5 W and (**b**) 1.0 W. This data is at the same location in each run where the maximum phase occurred (corresponding to the center of the heater). Current data did not vary significantly and thus the average of three runs is displayed.

**Figure 10:**
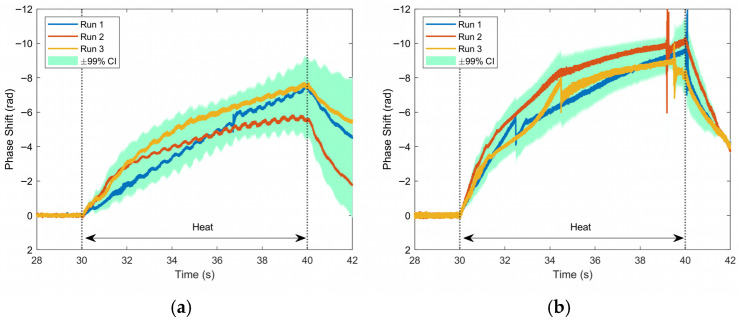
In-magnet test phase as a function of time for a single channel for three separate runs at the location of maximum phase change for each heating power: (**a**) 0.5 W and (**b**) 1.0 W, with heating being applied for 10 s in both cases. This data is at the same location as in Figure 8.

This final section will focus on in-magnet temperature detection using the coherent-phase OTDR technique. Figure 9 and Figure 10 shows graphs of phase as a function of time for the channel with the maximum phase change for the 0.5 and 1.0 W heater powers. Each of the three curves represents a different run under the same conditions, showing good repeatability. Figure 9 focuses on the period of each run where current is applied to the magnet; the average transport current profile is also shown in this plot. As current increases, self-induced Lorentz forces produce expansion and contraction at different locations on the magnet. The results of these are clearly seen in the phase response in Figure 9, in the charging, plateau, and discharging of the coil. These measurements of Lorentz force strain are verified by previous experiments using a different Rayleigh-based technique, OFDR [8]. In this previous study, alternating current was applied to the magnets under test, which resulted in a signal due to periodic Lorentz force strain. Due to the time-dependent, periodic nature of these signals, they can be filtered from temperature. However, the present study used a magnet operated with direct current and thus was not subjected to these periodic signals. In the plateau regions (20–60 s) at 45 A, phase was not completely constant, and these varying trends were linearly filtered when plotting Figure 10, allowing for a clearer distinction between the 0.5 and 1.0 W heater powers. There is a distinct relationship between phase and not only the time when heat is applied, but also the amount of heat applied. The total phase change accumulated during heating seems to be proportional to the heating power, on average −6.791 rad for the 0.5 W case and −9.231 rad for the 1.0 W case, over the three repeated measurements. In addition, there is a strong impulse as the heating is turned off, most clearly in the 1.0 W case.

**Figure 11:**
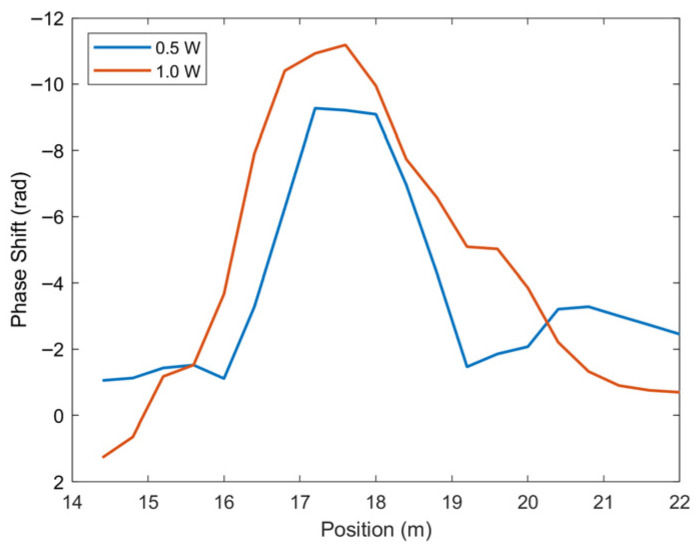
In-magnet spatial distribution of phase for Run 2 at each heater power and centered on the location of the heater. Strain and vibrational trends were filtered out by calculating the difference between phase at one second before the initial and final times of the heating period.

Figure 11 represents a spatial phase response for the second run of the 0.5 and 1.0 heating cases centered on the heater located on the small magnet coil. This data was filtered to remove the baseline phase response of the plateau, allowing for the response caused by the heater to be isolated. This spatial response confirms that results shown in Figure 10 occur over a noticeable distribution, centered on the location of the heater. This distribution also seems to be wider for the 1.0 W power, as more heat is imparted and dissipates farther into the magnet. The spatial plot also displays a specific feature of the coherent-phase ODTR sensing technique. To achieve high measurement rates over long distances, the sections of fiber length over which each response is measured must be large in comparison to other techniques like ODFR. The expected response for heating at one position at the edge of a coil would be period. However, this is not seen due to each channel averaging all changes over 2 m, which is much larger than the coil radius. Despite the large channel length, a phase response can still be resolved for heating powers of 0.5 and 1.0 W, and it is even possible to distinguish between the two. This correlation with the heater both temporally and spatially suggests that the introduction of heat was successfully detected by the system. Table 2 summarizes the measured sensitivity of phase to the heating signal for the two heater powers. Compared to the localized heater tests, this series of tests has less noise for the same power level. It is possible that this was related to the fact that the fiber was wound into the magnet coils and not in direct contact with liquid nitrogen. Overall, the ability to detect heating and show the difference between different heating power levels was demonstrated.

As the principal application of this sensing technique is fusion technology, radiation effects must be acknowledged. This study focuses on initial cryogenic characterization of the coherent-phase OTDR technique, but future work must investigate how the components of this technique are affected by defects and other effects of irradiation. In addition, all fibers are subject to radiation-induced attenuation (RIA), where point defects in the core and cladding of the fiber reduce light transmission. As RIA is dependent on distance, the long lengths of fiber required for quench detection in fusion magnets will intensify this issue. RIA can be mitigated by use of fiber chemistries and structures that are the most radiation-tolerant [43,44,45]. In addition, in situ, in operando processes like photobleaching can reduce the concentration of defects and thus recover transmission [20,46]. Specific effects of ionizing radiation on the coherent-phase OTDR technique have not yet been investigated and will be the focus of future studies.

## 4. Conclusions

This study characterized a novel distributed fiber optic diagnostic technique for cryogenic continuous monitoring within superconducting magnets of fusion reactors. Several laboratory experiments were carried out to evaluate this system’s abilities to quickly detect the localized heat associated with quench events at cryogenic temperatures. The system proved to have good temperature sensitivity for increases in temperature of a few degrees Kelvin. The technique demonstrated the capability to detect localized heating events, sufficiently tracking thermal transients over as small as 5 cm of fiber length, despite this being only 2.5% of the fiber channel length. Notably, this technique also showed temperature sensitivity in the range 20–30 K, which is where HTS-based fusion tokamaks will operate, with a temperature resolution of 0.9 K at 20 K (68% CI). However, there are some deviations from this monotonic linear trend, motivating future work investigating cross-sensitivities of this technique at cryogenic temperatures. These outcomes were demonstrated to be repeatable for both standalone fibers and fibers embedded in HTS magnet coils. Due to the wide range of frequencies captured, it is possible to differentiate long-term temperature trends from noise caused by strain and vibration signals. More work is needed in this area to evaluate the extent to which cross-sensitivities can be reduced or eliminated using real-time frequency domain analysis. Overall, the coherent-phase OTDR proved to be a promising method for the rapid detection of quench events in large HTS magnet systems.

## Figures and Tables

**Table 1:** Localized heater test statistics for 5 cm heater in nitrogen vapor.

Heater Power (W)	Temp. Change (K)	Phase Change (rad)	SNR (dB)	Temp. Sens. (rad/K)
0.5	10 (248)	70	20.7	7.0
1.0	35 (208)	200	17.3	5.7
5.0	170 (173)	900	36.9	5.3

**Table 2:** Dual magnet heating test statistics.

Heater Power (W)	Phase Change (rad)	SNR (dB)	Power Sens. (rad/W)
0.5	4	26.5	8.0
1.0	6	27.2	6.0

## Data Availability

The raw data supporting the conclusions of this article will be made available by the authors on request.
